# Supplementary material for: Perinatal and Demographic Risk Factors Associated with Autism Spectrum Disorder: A National Survey of Potential Predictors and Severity
Source: Healthcare (Basel). 2024 Oct 16;12(20):2057. doi: 10.3390/healthcare12202057 (PMC11507011; doi:10.3390/healthcare12202057)
Supplement: Supplementary file 1 [file healthcare-12-02057-s001.zip › healthcare-3189831-supplementary file S1.pdf]

UNIVERSITY OF WEST ATTICA
ΠΑΝΕΠΙΣΤΗΜΙΟ ΔΥΤΙΚΗΣ ΑΤΤΙΚΗΣ

INVITATION TO PARTICIPATE IN A SURVEY

You are invited to participate in a scientific research conducted by **Aikaterini Sousamli, PhD candidate at the Department of Midwifery of the University of Athens**. The title of the Research Program in which you are invited to participate is**: Study of perinatal factors related to the occurrence of ASD in children**. Your participation is voluntary. You can take as much time as you need to read **the questionnaire update page. The completion and return of the questionnaire constitutes consent to participate in this survey.** You can also decide to discuss it with your family or friends. The research has been approved by the Ethics Committee of the University of West Attica (Reference Number: 29346/08-04-2024).

PURPOSE OF THE RESEARCH

We are asking you to participate in this research because its purpose is to investigate the prenatal and perinatal risk factors associated with the occurrence of Autism Spectrum Disorders in children. **The completion and return of the questionnaire constitutes consent to participate in this survey.** The survey is addressed to mothers of children, aged 4 to 7 with ASD, who speak and understand the Greek language. You have been invited to participate in this research because it will be understood the impact of common perinatal risk factors on mothers who have given birth to children with ASD, to help support and improve social care and mental health care services for the mental health and well-being of mothers/ parents/ caregivers of children with ASD, in developing programs, interventions and resources for these families.

PARTICIPATION AND WITHDRAWAL

Your participation is **voluntary** and necessary to conduct this study. You may refuse to participate without any reason or excuse. You can change your mind at any time and withdraw from the survey without any reason or excuse and without any consequence to you. In this case you can request that we delete your data or stop a part of the processing. There are no right or wrong answers, but we are interested in recording your own personal experience, so please answer in all honesty. It only takes a few minutes of your time to complete the questionnaire. Your answers are **anonymous** and **confidential, no one has access to the data other than the researchers** and the results obtained will be used for the statistical analysis of the specific survey. Please answer all questions and make sure they are completed at the end of the process.

POTENTIAL RISKS

Your participation in the survey does not entail any particular risk or financial burden for you. If you experience discomfort while answering specific questions, please do not hesitate to ask for them to be omitted.

POTENTIAL BENEFITS FOR INDIVIDUALS AND SOCIETY

There are no specific benefits to participating in this survey. The findings will understand the impact of common perinatal risk factors on mothers who have given birth to children with ASD, to help support and improve social care services and care for the mental health and well-being of mothers/ parents/ caregivers of children with ASD, in developing programs, interventions and resources for these families. The isolation of families (especially mothers) can be devastating to the upbringing of a child with autism.

The prevalence of ASD has increased significantly over the past 20 years. Population growth and rising rehabilitation costs for people with ASD have become a serious social problem. Our results will probably support the theory that the occurrence of ASD is multifactorial and some risk factors to be studied may indicate statistical significance. Through the results of this thesis, perinatal care professionals, mainly midwives, will get to know risk factors concerning the mother prenatally and perinatally with the ultimate goal of controlling the ever-increasing prevalence of the disease and supporting parents and families with a child suffering from ASD.

Lack of knowledge from health professionals can be a barrier to autism diagnosis and support. It should be noted that mental health support during pregnancy is necessary to prevent or reduce stress in pregnant women at high risk for ASD. The cost to families with ASD is so high that the benefits of preventing even one incident of ASD are crucial.

COMPENSATION FOR PARTICIPATION

There is no fee for your participation in the survey, nor will you incur any costs.

POTENTIAL CONFLICT OF INTEREST

According to the statement of the researchers of the study, there is no conflict of interest.

CONFIDENTIALITY

In this survey, your personal data will be used exclusively for research, scientific and statistical purposes. Your data will be anonymized (encrypted).) The database of research participants will be kept in a secure place with access only by the research team. Any information obtained in connection with this research that could personally identify you will remain confidential and will only be disclosed with your permission or as permitted by law. Information that personally identifies you will be kept separate from other data about you. The responsible researcher will keep your personal data in writing and in an electronic file, within her office, within a period of two years, which can be

made available to the Supervisory Authority upon request. Your data will be processed based on your express consent, which you provide to us through this form. Your anonymity is ensured throughout the research and when publishing the results in conferences, scientific journals, etc.

IDENTITY OF RESEARCHERS

For any questions regarding the scientific research, you can contact the responsible researcher Aikaterini Sousamli, PhD candidate, Department of Midwifery, University of West Attica, asousamli@uniwa.gr, 6943477660, as well as the Responsible Supervising Professor of Doctoral Research for questions about the personal data concerning you, Antigoni Sarantaki, Associate Professor of the Department of Midwifery, University of West Attica, esarantaki@uniwa.gr, 6977613848. If you have any questions or concerns about the survey, please do not hesitate to contact the researcher.

Submission of Complaints

For any complaint regarding the conduct of the research, you can contact the Research Ethics Committee of the University of West Attica (ethics@uniwa.gr). For any complaint regarding the management of your personal data, you can also contact the Personal Data Protection Officer of the University of West Attica, Mr. D. Bletsas. (d.mpletsas@uniwa.gr). If your problem is not resolved, you may contact the Personal Data Protection Authority by filling in the relevant form on its website (complaints@dpa.gr).

DECLARATION OF CONSENT	Yes	No
I, the undersigned, declare that I have been fully informed about the terms of my participation in the survey and the processing of my personal data.<br>I provide my express consent for my participation in the research and the processing of my personal data mentioned above.<br>I have been informed that I can withdraw my consent at any time.		

Participant code:	(To be completed by the Data Controller)
Name of researcher:	Aikaterini Sousamli
Date:	1/ 4/ 2024
Signature of researcher:	

QUESTIONNAIRE

A. Demographics

A1. Mother's year of birth [_____]

A2. Family Type

a. Two parents living together in the same house □ 0

b. Two parents living separately □ 1

c. Single-parent family (only with one parent) □ 2

d. Adoptive/ foster parents □ 3

A3. Educational level of the mother:

a. Primary education (Primary) □

b. Secondary education (Junior High school - High school) □

c. Higher education (University education) □

d. Master's degree holder □

e. Holder of PhD □

A4. Year of birth of father [__][__][__][__]

A5. Educational level of the father:

a. Primary education (Primary) □

b. Secondary education (Junior High school - High school□

c. Higher education (University education) □

d. Master's degree holder □

e. Holder of PhD □

A6. Annual total family income:

Under 10,000€	10,001-20.000 €	20,001-40,000€	40,001-60.000€	over 60,000€
□ 0	□ 1	□ 2	□ 3	□ 4

A7. Place of residence of the family (now),

....

A8. Year of birth of the child with ASD: [_____]

A9. Gender of child with ASD: Boy □ Girl □ Other □

A10. Year of ASD diagnosis: [____]

A11. Functionality of a child with ASD: High □ Medium □ Low □

A12. Please indicate the ages and genders of all siblings of the child:

1. ☐ Male ☐ Female □ Other Age [______]
2. ☐ Male ☐ Female □ Other Age [_____]
3. ☐ Male ☐ Female □ Other Age [__][__]
4. ☐ Male ☐ Female □ Other Age [______]
5. ☐ Male ☐ Female □ Other Age [______]

A13. Birth order of the child with ASD

Is the 1st child	2nd child	3rd child	4th child	5th child	Other
□ 0	□ 1	□ 2	□ 3	□ 4	□ 5

B. Clinical characteristics

B1. Does another child have neurodevelopmental difficulties or autism?

**YES**	**NO**
□ 0	□ 1

B2. In the family history of the mother, are there blood relatives with autism, developmental disorders, seizures or depression, or anxiety disorder?

**YES**	**NO**
□ 0	□ 1

B3. In the father's family history, are there blood relatives with autism, developmental disorders, seizures or depression, anxiety disorder?

**YES**	**NO**
□ 0	□ 1

B4. When you became pregnant, please note if any of the following happened:

a. Have you come into contact with chemicals/ pesticides? Yes □ No □

b. Did you come into contact with loud noise every day? Yes □ No □

c. Did you live near a PPC substation**;** Yes □ No □

B5. What was your weight in kilograms **before** you became pregnant? : [__][__][__]

During pregnancy

Mother:

B6. Maternal age **when pregnant** (in years): [__][__]

B7. What was your weight and height at the end of pregnancy?

a. Weight in kilograms [_______]

b. Height in centimeters [_____]

B8. Did you smoke during pregnancy?

YES	NO
□ 0	□ 1

B9. Did you experience any of the following conditions during the pregnancy of your child with ASD?

	YES	NO
a. Gestational diabetes	□ 1	□ 2
b. Hyperemesis (a lot of vomiting)	□ 1	□ 2
c. Vaginal bleeding (blood from the vagina)	□ 1	□ 2
If yes, in which trimester of pregnancy [__]		
d. Viral or bacterial infection	□ 1	□ 2
If yes, in which week of pregnancy [__][__]		
e. High blood pressure	□ 1	□ 2
F. Preeclampsia/ eclampsia	□ 1	□ 2

Father:

B10. Father's Age, When You Became Pregnant, Years: [__][__]

C. History of birth / delivery

C1. At what gestational age did you give birth (in weeks)? [__][__]

C2. How many hours did your birth last (total)? [__] [__]

C3. Did you give birth by normal birth? □ Yes □ No

C4. Did you give birth vaginally but with a ventouse (vacuum cup)? □ Yes □ No

C5. Did you give birth by caesarean section? □ Yes □ No

C6. Was your labor induced with medications (oxytocin)? □ Yes □ No □ I don't know

C7. Have you been given any other medicines during childbirth (e.g. pethidine)?

□ Yes □ No □ I don't know

If so, which one..

C8. Did your baby <u>cry right after birth</u>? □ Yes □ No □ I don't remember

C9. Birth weight of the child (in grams) [_____]

D. Postpartum factors/ Postpartum

Neonate:

D1. Did the newborn have an infection after childbirth? □ Yes □ No

D2. Did you breastfeed exclusively? □ Yes □ No

D3. Mixed feeding of the newborn (breastfeeding and formula-foreign milk)? □ Yes □ No

D4. Did you use exclusively formula (foreign milk)? □ Yes □ No

E. Social history

Please tick the ☑ box that <u>most closely matches your answer(s)</u> in <u>each question</u> below.

E1. How adequate do you feel as a parent?

Not at all	Slightly	Enough	Very
□ 0	□ 1	□ 2	□ 3

E2. Regarding your child's future, which of the following feelings do you feel most often?

	Never	Sometimes	Often	Continuously
a. Anguish	□ 0	□ 1	□ 2	□ 3
b. Protectiveness	□ 0	□ 1	□ 2	□ 3
c. Grief	□ 0	□ 1	□ 2	□ 3
d. Happiness	□ 0	□ 1	□ 2	□ 3
e. Oppression	□ 0	□ 1	□ 2	□ 3
f. Despair	□ 0	□ 1	□ 2	□ 3
g. Satisfaction	□ 0	□ 1	□ 2	□ 3
h. Safety	□ 0	□ 1	□ 2	□ 3

i. Other, please specify..............................	□ 0	□ 1	□ 2	□ 3

Q3. Have you been concerned about what others think of your child?

NEVER	RARE	SOMETIMES	OFTEN	CONTINUOUSLY
□ 0	□ 1	□ 2	□ 3	□ 4

Q4. Have you felt ashamed of your child?

NEVER	RARE	SOMETIMES	OFTEN	CONTINUOUSLY
□ 0	□ 1	□ 2	□ 3	□ 4

Thank you very much for your participation!!!
