# Supplementary material for: Perinatal and Demographic Risk Factors Associated with Autism Spectrum Disorder: A National Survey of Potential Predictors and Severity
Source: Healthcare (Basel). 2024 Oct 16;12(20):2057. doi: 10.3390/healthcare12202057 (PMC11507011; doi:10.3390/healthcare12202057)
Supplement: Supplementary file 1 [file healthcare-12-02057-s001.zip › healthcare-3189831-supplementary.pdf]

# Supplementary Material


**Table S1. Descriptive Statistics of Continuous Variables** ............................................. 2

**Table S2. Descriptive Statistics of Sociodemographic Categorical Variables** ................. 3

**Table S3. Descriptive Statistics of ASD-Related Categorical Variables** ......................... 4

**Table S4. Descriptive Statistics of Peri- and Postnatal Categorical Variables** ................ 5

**Table S5. Comparison of Continuous Variables by Functionality of Child with ASD** .... 7

**Table S6. Group Descriptives of Continuous Variables by Functionality of Child with ASD** ........................................................................................................................ 8

**Table S7. Collinearity Statistics of Binominal Logistic Regression** ................................ 9

**Figure S1. The visualization of the Chi-square test results for the Current Family Residence** ........................................................................................................................10

**Figure S2. Cut-Off Plot of the Binominal Logistic Regression Prediction Model** ..........11

**Figure S3. ROC Curve of the Binominal Logistic Regression Prediction Model** ...........12

**Table S1.** **Descriptive Statistics of Continuous Variables.**

| Variable | N | M | Mdn | SD | Min | Max |
|---|---|---|---|---|---|---|
| Mother's Age | 517 | 40.2 | 40 | 5.42 | 21 | 63 |
| Father's Age | 517 | 43.3 | 43 | 6.19 | 24 | 70 |
| Age of Child with ASD | 517 | 6.89 | 6 | 3.94 | 1 | 28 |
| Age at ASD Diagnosis | 517 | 3.47 | 3 | 2.29 | 0 | 24 |
| Pre-pregnancy Weight (kg) | 517 | 67.2 | 63 | 33.4 | 34 | 761 |
| Mother's Age at Conception | 517 | 32.9 | 33 | 4.95 | 17.0 | 65.0 |
| Weight at End of Pregnancy | 517 | 80.4 | 78 | 29.1 | 49.0 | 651 |
| Height at End of Pregnancy (cm) | 517 | 164 | 165 | 16.1 | 1.52 | 188 |
| Father's Age at Conception | 517 | 36.0 | 36 | 5.61 | 20.0 | 65.0 |
| Gestational Age at Birth (weeks) | 504 | 37.9 | 38 | 2.74 | 9.00 | 42.0 |
| Duration of Labor (hours) | 517 | 4.88 | 2 | 6.14 | 0 | 48 |
| Duration of Labor (minutes) | 517 | 293 | 120 | 368 | 0 | 2880 |
| Birth Weight of Child (grams) | 513 | 3089 | 3150 | 744 | 2.90 | 4850 |

Note: M = Mean, Mdn = Median, SD = Standard Deviation, Min = Minimum, Max = Maximum.

**Table S2. Descriptive Statistics of Sociodemographic Categorical Variables.**

| Variable | Counts | % of Total | Cumulative % |
|---|---|---|---|
| **Family Type** | | | |
| Two parents living together in the same house | 466 | 90.1 % | 90.1 % |
| Two parents living separately | 26 | 5.0 % | 95.2 % |
| Adoptive/foster parents | 1 | 0.2 % | 95.4 % |
| Single-parent family (only one parent) | 24 | 4.6% | 100.0% |
| **Mother's Educational level** | | | |
| Secondary education (Middle School - High School) | 131 | 25.3 % | 25.3 % |
| PhD holder | 13 | 2.5 % | 27.9 % |
| Master's degree holder | 147 | 28.4 % | 56.3 % |
| Primary education (Elementary School) | 1 | 0.2 % | 56.5 % |
| Tertiary education (University education) | 225 | 43.5 % | 100.0% |
| **Father's Educational level** | | | |
| Secondary education (Middle School - High School) | 246 | 47.6 % | 47.6 % |
| PhD holder | 12 | 2.3 % | 49.9 % |
| Master's degree holder | 69 | 13.3 % | 63.2 % |
| Primary education (Elementary School) | 9 | 1.7 % | 65.0 % |
| Tertiary education (University education) | 181 | 35.0% | 100.0% |
| **Annual Family Income** | | | |
| €10,001-20,000 | 196 | 38.1% | 38.1% |
| €20,001-40,000 | 217 | 42.1% | 80.2% |
| €40,001-60,000 | 35 | 6.8% | 87.0% |
| Below €10,000 | 44 | 8.5% | 95.5% |
| Above €60,000 | 23 | 4.5% | 100.0% |
| **Current Family Residence** | | | |
| Thessaly | 63 | 12.2 % | 12.2 % |
| North Aegean | 15 | 2.9 % | 15.1 % |
| Attica | 189 | 36.6 % | 51.7 % |
| Central Macedonia | 69 | 13.4 % | 65.1 % |
| Crete | 26 | 5.0 % | 70.2 % |
| Eastern Macedonia and Thrace | 27 | 5.2 % | 75.4 % |
| South Aegean | 15 | 2.9 % | 78.3 % |
| Western Greece | 34 | 6.6 % | 84.9 % |
| Ionian Islands | 10 | 1.9 % | 86.8 % |
| Western Macedonia | 8 | 1.6 % | 88.4 % |
| Peloponnese | 26 | 5.0 % | 93.4 % |
| Central Greece | 21 | 4.1 % | 97.5 % |
| Epirus | 13 | 2.5% | 100.0% |

**Table S3.** **Descriptive Statistics of ASD-Related Categorical Variables.**

| Variable | Counts | % of Total | Cumulative % |
|---|---|---|---|
| **Functionality of Child with ASD** | | | |
| Low/Moderate | 195 | 37.7 % | 37.7 % |
| High | 322 | 62.3% | 100.0% |
| **Gender of Child with ASD** | | | |
| Boy | 419 | 81.0% | 81.0% |
| Girl | 98 | 19.0% | 100.0% |
| **Birth Order of Child with ASD** | | | |
| 1st child | 348 | 67.3 % | 67.3 % |
| 2nd child | 137 | 26.5 % | 93.8 % |
| 3rd child | 27 | 5.2 % | 99.0 % |
| 4th child | 2 | 0.4 % | 99.4 % |
| 5th child | 2 | 0.4 % | 99.8 % |
| Other | 1 | 0.2% | 100.0% |
| **Other Children with Neurodevelopmental Difficulties or Autism** | | | |
| No | 462 | 89.4 % | 89.4 % |
| Yes | 55 | 10.6 % | 100.0% |
| **Mother's Family History with Autism, Developmental Disorders, Epileptic Seizures, Depression, or Anxiety Disorder** | | | |
| No | 350 | 67.7 % | 67.7 % |
| Yes | 167 | 32.3% | 100.0% |
| **Father's Family History with Autism, Developmental Disorders, Epileptic Seizures, Depression, or Anxiety Disorder** | | | |
| No | 315 | 60.9 % | 60.9 % |
| Yes | 202 | 39.1% | 100.0% |

**Table S4.** **Descriptive Statistics of Peri- and Postnatal Categorical Variables.**

| | Counts | % of Total | Cumulative % |
|---|---|---|---|
| **Contact with Chemicals/Pesticides** | | | |
| No | 486 | 94.0% | 94.0% |
| Yes | 31 | 6.0% | 100.0% |
| **Exposure to loud noise daily** | | | |
| No | 445 | 86.1% | 86.1% |
| Yes | 72 | 13.9% | 100.0% |
| **Living near a Public Power Corporation (PPC) substation** | | | |
| No | 480 | 92.8% | 92.8% |
| Yes | 37 | 7.2% | 100.0% |
| **Smoke during pregnancy** | | | |
| No | 446 | 86.3% | 86.3% |
| Yes | 71 | 19.0% | 100.0% |
| **Gestational Diabetes** | | | |
| No | 420 | 81.2% | 81.2% |
| Yes | 97 | 18.8% | 100.0% |
| **Hyperemesis (severe vomiting)** | | | |
| No | 449 | 86.8% | 86.8% |
| Yes | 68 | 13.2% | 100.0% |
| **Viral or Bacterial Infection** | | | |
| No | 453 | 87.6% | 87.6% |
| Yes | 64 | 12.4% | 100.0% |
| **Viral or Bacterial Infection during the pregnancy of the child with ASD** | | | |
| No | 445 | 86.1 % | 86.1 % |
| Don't remember | 1 | 0.2% | 86.3% |
| Yes | 71 | 13.7 % | 100.0 % |
| **High Blood Pressure** | | | |
| No | 476 | 92.1% | 92.1% |
| Yes | 41 | 7.9% | 100.0% |
| **Preeclampsia/Eclampsia** | | | |
| No | 497 | 96.1% | 96.1% |
| Yes | 20 | 3.9% | 100.0% |
| **Vaginal Bleeding** | | | |
| No | 409 | 79.1% | 79.1% |
| Yes | 108 | 20.9% | 100.0% |
| **Vaginal bleeding during the pregnancy of the child with ASD (trimester)** | | | |
| 1st trimester | 83 | 16.1% | 16.1% |
| 2nd trimester | 19 | 3.7% | 19.7% |
| 3rd trimester | 19 | 3.7% | 23.4% |

| | Counts | % of Total | Cumulative % |
|---|---|---|---|
| No | 396 | 76.6% | 100.0% |
| **Normal Delivery** | | | |
| No | 325 | 62.9% | 62.9% |
| Yes | 192 | 37.1% | 100.0% |
| **Vaginal Delivery with Vacuum Extraction** | | | |
| No | 486 | 94.0% | 94.0% |
| Yes | 31 | 6.0% | 100.0% |
| **Cesarean Section** | | | |
| No | 192 | 37.1% | 37.1% |
| Yes | 325 | 62.9% | 100.0% |
| **Labor Induced with Medication** | | | |
| No | 322 | 62.3% | 62.3% |
| Don't know | 49 | 9.5% | 71.8% |
| Yes | 146 | 28.2% | 100.0% |
| **Other Medications During Labor** | | | |
| No | 213 | 41.2% | 41.2% |
| Don't know | 276 | 53.4% | 94.6% |
| Yes | 28 | 5.4% | 100.0% |
| **Baby Cried Immediately After Birth** | | | |
| No | 49 | 9.5% | 9.5% |
| Don't remember | 44 | 8.5% | 18.0% |
| Yes | 424 | 82.0% | 100.0% |
| **Newborn Developed Infection After Birth** | | | |
| No | 479 | 92.6% | 92.6% |
| Yes | 38 | 7.4% | 100.0% |
| **Exclusively Breastfed** | | | |
| No | 293 | 56.7% | 56.7% |
| Yes | 224 | 43.3% | 100.0% |
| **Mixed Feeding of Newborn (Breastfeeding and Formula)** | | | |
| No | 274 | 53.0% | 53.0% |
| Yes | 243 | 47.0% | 100.0% |
| **Exclusive Formula Feeding** | | | |
| No | 400 | 77.4 % | 77.4 % |
| Yes | 117 | 22.6% | 100.0% |

**Table S5.** **Comparison of Continuous Variables by Functionality of Child with ASD.**

| | | Statistic | df | p |
|---|---|---|---|---|
| Mother's Age | Student's t | 2.214 | 515 | **0.027** |
| Father's Age | Student's t | 1.279 | 515 | 0.202 |
| Age of Child with ASD | Student's t | 0.127 | 515 | 0.899 |
| Age at ASD Diagnosis | Student's t | -5.431 | 515 | < .001 |
| Pre-pregnancy Weight (kg) | Student's t | -0.955 | 515 | 0.340 |
| Mother's Age at Conception | Student's t | 2.443 | 515 | 0.015 |
| Weight at End of Pregnancy (kg) | Student's t | -1.114 | 515 | 0.266 |
| Height at End of Pregnancy (cm) | Student's t | 0.712 | 507 | 0.477 |
| Father's Age at Conception | Student's t | 1.748 | 512 | 0.081 |
| Gestational Age at Birth (weeks) | Student's t | -1.855 | 502 | 0.064 |
| Duration of Labor (hours) | Student's t | -1.227 | 515 | 0.220 |
| Duration of Labor (minutes) | Student's t | -1.227 | 515 | 0.220 |
| Birth Weight of Child (grams) | Student's t | 0.177 | 511 | 0.860 |

Note. H $\mu$ Functionality of Child with ASD = Low/Moderate $\neq$ $\mu$ Functionality of Child with ASD = High

**Table S6. Group Descriptives of Continuous Variables by Functionality of Child with ASD.**

| | Group | N | Mean | Median | SD | SE |
|---|---|---|---|---|---|---|
| Mother's Age | Low/Moderate | 195 | 40.86 | 41.00 | 5.38 | 0.385 |
| | High | 322 | 39.78 | 40.00 | 5.41 | 0.302 |
| Father's Age | Low/Moderate | 195 | 43.78 | 44.00 | 5.38 | 0.385 |
| | High | 322 | 43.06 | 42.00 | 6.62 | 0.369 |
| Age of Child with ASD | Low/Moderate | 195 | 6.92 | 6.00 | 4.05 | 0.290 |
| | High | 322 | 6.87 | 6.00 | 3.87 | 0.216 |
| Age at ASD Diagnosis | Low/Moderate | 195 | 2.78 | 3.00 | 1.46 | 0.105 |
| | High | 322 | 3.89 | 3.00 | 2.59 | 0.144 |
| Pre-pregnancy Weight (kg) | Low/Moderate | 195 | 65.39 | 62.00 | 12.54 | 0.898 |
| | High | 322 | 68.29 | 63.00 | 41.14 | 2.293 |
| Mother's Age at Conception | Low/Moderate | 195 | 33.63 | 33.00 | 5.05 | 0.362 |
| | High | 322 | 32.53 | 32.00 | 4.86 | 0.271 |
| Weight at End of Pregnancy (kg) | Low/Moderate | 195 | 78.53 | 77.00 | 13.36 | 0.957 |
| | High | 322 | 81.48 | 78.00 | 35.37 | 1.971 |
| Height at End of Pregnancy (cm) | Low/Moderate | 190 | 164.89 | 165.00 | 13.28 | 0.964 |
| | High | 319 | 163.84 | 165.00 | 17.54 | 0.982 |
| Father's Age at Conception | Low/Moderate | 194 | 36.56 | 37.00 | 5.17 | 0.371 |
| | High | 320 | 35.67 | 35.00 | 5.85 | 0.327 |
| Gestational Age at Birth (weeks) | Low/Moderate | 190 | 37.61 | 38.00 | 3.15 | 0.229 |
| | High | 314 | 38.08 | 38.00 | 2.44 | 0.138 |
| Duration of Labor (hours) | Low/Moderate | 195 | 4.46 | 2.00 | 6.51 | 0.466 |
| | High | 322 | 5.14 | 3.00 | 5.90 | 0.329 |
| Duration of Labor (minutes) | Low/Moderate | 195 | 267.38 | 120.00 | 390.47 | 27.962 |
| | High | 322 | 308.39 | 180.00 | 354.17 | 19.737 |
| Birth Weight of Child (grams) | Low/Moderate | 195 | 3096.46 | 3180.00 | 670.59 | 48.022 |
| | High | 318 | 3084.49 | 3150.00 | 786.47 | 44.103 |

**Table S7.** **Collinearity Statistics of Binominal Logistic Regression.**

| | VIF | Tolerance |
|---|---|---|
| Mother's Age | 1.49 | 0.671 |
| Age at ASD Diagnosis | 1.04 | 0.961 |
| Mother's Age at Conception | 1.45 | 0.692 |
| Annual family income | 1.03 | 0.969 |
| Birth Order of Child with ASD | 1.02 | 0.983 |
| Mother's Family History with Autism, Developmental Disorders, Epileptic Seizures, Depression, or Anxiety Disorder | 1.01 | 0.990 |
| Hyperemesis (severe vomiting) | 1.02 | 0.980 |
| Viral or Bacterial Infection | 1.03 | 0.969 |
| Vaginal Bleeding | 3.01 | 0.332 |
| Vaginal Bleeding during the pregnancy of the child with ASD (trimester) | 1.46 | 0.685 |
| Baby Cried Immediately After Birth | 1.02 | 0.976 |

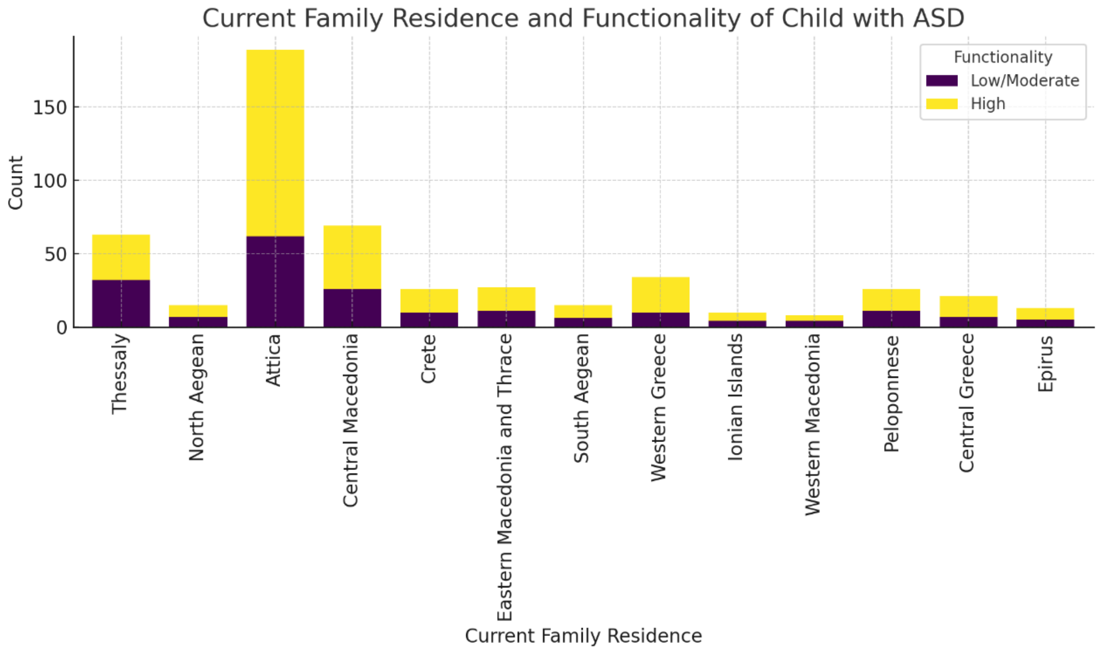


**Figure S1. The visualization of the Chi-square test results for the Current Family Residence.**

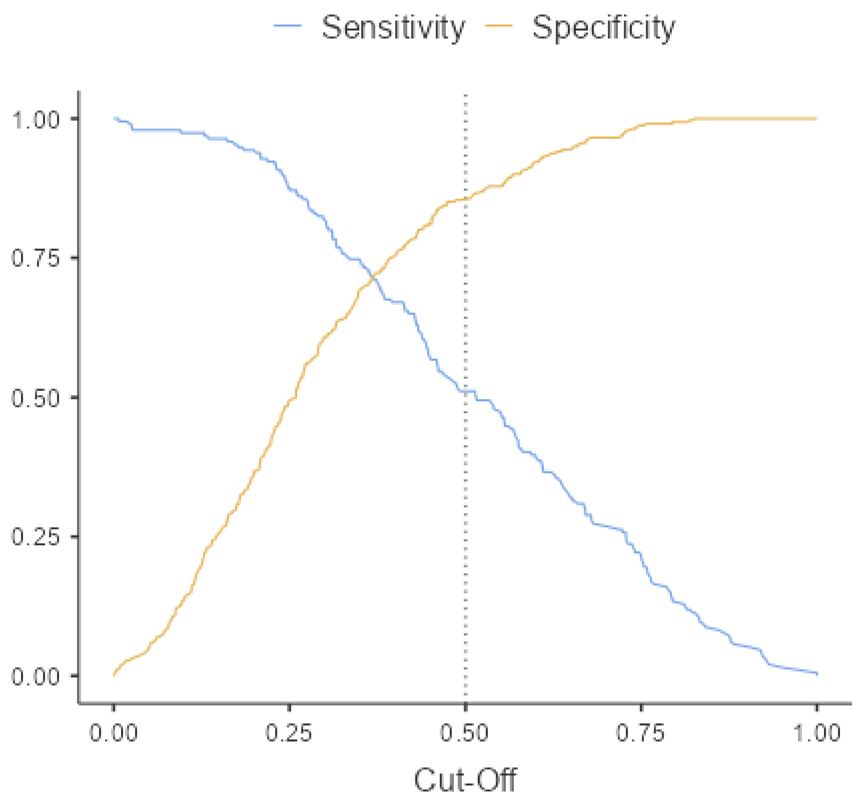


**Figure S2. Cut-Off Plot of the Binominal Logistic Regression Prediction Model.**

Classification Table – ...

| | Predicted | | |
|---|---|---|---|
| Observed | High | Low/Moderate | % Correct |
| High | 275 | 46 | 85.7 |
| Low/Moderate | 95 | 99 | 51.0 |

Note. The cut-off value is set to 0.5

Predictive Measures

| Accuracy | Specificity | Sensitivity | AUC |
|---|---|---|---|
| 0.726 | 0.857 | 0.510 | 0.790 |

Note. The cut-off value is set to 0.5

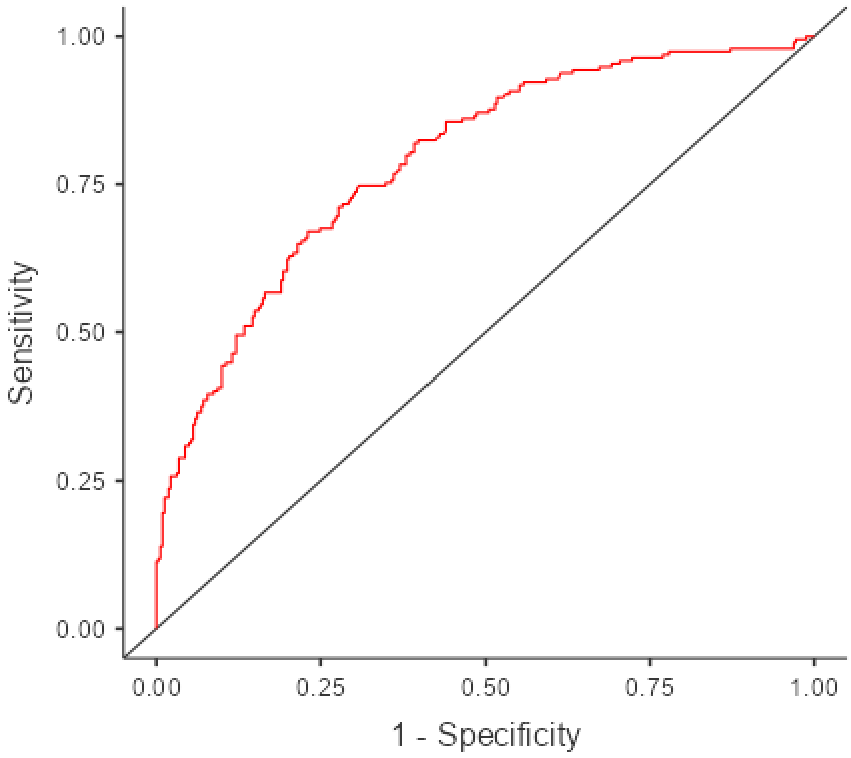


**Figure S3. ROC Curve of the Binominal Logistic Regression Prediction Model.**
